# Acupuncture as Add-On Treatment of the Positive, Negative, and Cognitive Symptoms of Patients with Schizophrenia: A Systematic Review

**DOI:** 10.3390/medicines5020029

**Published:** 2018-03-30

**Authors:** Maurits van den Noort, Sujung Yeo, Sabina Lim, Sook-Hyun Lee, Heike Staudte, Peggy Bosch

**Affiliations:** 1 Research Group of Pain and Neuroscience, Kyung Hee University, Seoul 130-701, Korea; sh00god@khu.ac.kr; 2 Brussels Institute for Applied Linguistics, Vrije Universiteit Brussel, 1050 Brussels, Belgium; 3 College of Oriental Medicine, Sang Ji University, Wonju 26339, Korea; pinkteeth@hanmail.net; 4 Psychiatric Research Group, LVR-Klinik Bedburg-Hau, 47511 Bedburg-Hau, Germany; Heike.Staudte@lvr.de (H.S.); p.bosch@donders.ru.nl (P.B.); 5 Donders Institute for Brain, Cognition and Behaviour, Radboud University, 6525 Nijmegen, The Netherlands

**Keywords:** schizophrenia, psychosis, sleep, sleep disturbances, sleep disorders, integrative medicine, acupuncture, add-on therapy

## Abstract

**Background:** Schizophrenia is a severe psychiatric disorder that has a large impact on patients’ lives. In addition to Western medicine, the use of additional treatments, such as acupuncture, in treating the positive, negative, and cognitive symptoms is increasing. **Methods:** We conducted a systematic review on the use of acupuncture as an add-on treatment for patients with schizophrenia that are in regular care, with a special focus on the treatment of the often accompanying sleep disorders. In this study, we searched the Medline, ScienceDirect, Cochrane Library, Scopus, and ERIC databases with a cut-off date of 31 December 2017, thereby following the guidelines of the Preferred Reporting Items for Systematic Reviews and Meta-analysis (PRISMA) protocol. **Results:** Our search resulted in 26 eligible studies with 1181 patients with schizophrenia who received acupuncture treatment. Most studies showed limited evidence for the use of acupuncture as add-on therapy in the treatment of the positive, negative, and cognitive symptoms, but beneficial effects have been reported in the treatment of the accompanying sleep disorders. **Conclusions:** Limited evidence was found for the use of acupuncture as add-on therapy in the treatment of patients with schizophrenia; however, positive results were found in the treatment of sleep disorders, but this result needs to be confirmed in large, randomized, controlled trials.

## 1. Introduction

Schizophrenia is a severe psychiatric disorder with a worldwide prevalence estimated somewhere between 0.3% and 0.7% [1]. More men than women are diagnosed with schizophrenia [2], with a male/female ratio of 1.4 to 1 [3]. Furthermore, the onset of the disorder is frequently reported to be several years earlier in men than in women [4]. For instance, in a study by Castle and colleagues [5], the peak age of onset of the disorder was found to be between 20 and 28 years of age for men compared to 26 and 32 years of age for women. In addition, the incidence of schizophrenia was found to be higher in cities compared to the countryside [6], and this seems to be especially the case for men [6].

Patients with schizophrenia suffer from positive symptoms, negative symptoms, and cognitive symptoms [7]. The positive symptoms refer to symptoms that are visible in patients with schizophrenia, for instance auditory, visual, olfactory, gustatory, or tactile hallucinations, and delusions, but are not present in healthy individuals [8]. On the contrary, the negative symptoms are symptoms that are present in healthy individuals, but are not visible in patients with schizophrenia. Here, one can think of the following examples: flat affect, inability to experience pleasure, emotional withdrawal, sleep problems, active social avoidance, and lack of motivation [9]. Finally, the cognitive symptoms refer to the problems that patients with schizophrenia experience in the cognitive domain, for instance, problems with (working) memory, focused and sustained attention, and problem solving [10]. The positive symptoms appear during psychotic episodes while the negative and cognitive symptoms are often already visible before the first psychotic episode [11]. That some symptoms interfere with each other is important to note; for instance, many patients with schizophrenia suffer from sleep problems, and as a result, they are tired and perform worse on cognitive tasks. Finally, the positive, negative, and cognitive symptoms have a large and often long-term impact on the patient’s life, and about ten percent of patients with schizophrenia eventually commit suicide [12].

The use of medications is still the cornerstone of disease management in patients with schizophrenia [13]. First- or second-generation antipsychotics are often prescribed [14]. However, despite their successes in the treatment of schizophrenia, several adverse effects exist, such as an increased risk of diabetes [15], extrapyramidal side-effects [16], tardive dyskinesia [17], weight gain [18], metabolic changes [19], and high drop-out rates [20]. Moreover, that large inter-individual differences exist in responses to the (long-term) use of antipsychotics is important to note [21].

Because of the above factors, the use of add-on therapies in the treatment of patients with schizophrenia is increasing. Nowadays, many intervention techniques are available, such as psychotherapy [22], social skills training [23], sleep training [24], family therapy [25], and vocational rehabilitation [26], but with different rates of success. Here, we will focus on one specific treatment technique, namely, acupuncture [27]. Although acupuncture has a long tradition in Eastern medicine in the treatment of patients with schizophrenia, it is a relatively new add-on treatment technique in the West, and many questions and uncertainties exist about its effects. Can acupuncture be safely applied to such a vulnerable patient group? Can acupuncture be beneficial as an add-on treatment technique in patients with schizophrenia, and if this is indeed the case, does it affect mostly the positive, negative, or cognitive symptoms?

The aim of the present study is to provide an overview of what has been done and what is known to date about the use of acupuncture as an add-on treatment for patients with schizophrenia. The focus will be on the safety issue of using acupuncture in this vulnerable patient group and on the treatment of the characteristic positive, negative, and cognitive symptoms of the disorder and the accompanying sleep disorders. We expect to find that acupuncture can be safely used in the treatment of schizophrenia. Moreover, we hypothesize that acupuncture will be able to treat the positive and negative symptoms of those patients, but only to a certain degree. Finally, we expect to find that acupuncture will alleviate the sleep disorders and the cognitive symptoms of patients with schizophrenia.

## 2. Materials and Methods

### 2.1. Search Strategies

We conducted a systematic review (registration number: NTR3132) on the use of acupuncture as an add-on treatment for patients with schizophrenia that are in regular care, with a special focus on the treatment of the often accompanying sleep disorders. In this study, we searched the Medline (https://www.ncbi.nlm.nih.gov/pubmed/), ScienceDirect (https://www.sciencedirect.com/), Cochrane Library (http://www.cochranelibrary.com/), Scopus (https://www.elsevier.com/solutions/scopus), and ERIC (https://eric.ed.gov/) databases with a cut-off date of 31 December 2017, thereby following the guidelines of the Preferred Reporting Items for Systematic Reviews and Meta-analysis (PRISMA) protocol [28]. We used the following combinations of keywords in our search: “schizophrenia” and “acupuncture”, “psychosis” and “acupuncture”, “schizophrenia” and “scalp acupuncture”, “psychosis” and “scalp acupuncture” and “auditory hallucination” and “acupuncture”. Only studies that used (mechanical) acupuncture in the treatment of patients with schizophrenia were selected in our review; studies involving other modalities, such as electro-acupuncture [29], laser-acupuncture [30], and acupressure [31] were excluded to be able to compare the study results. We also included studies that had been published in languages other than English (e.g., Chinese, Japanese, etc.), as long as the abstract had been published in English.

### 2.2. Study Selection and Data extraction

Two authors (H.S. and P.B.) independently searched the Medline, ScienceDirect, Cochrane Library, Scopus, and ERIC databases. The study selection and data extraction were independently performed by two different authors (M.N. and S.Y.). The extracted data consisted of the following information: the authors and the title of study, the journal in which the study had been published, the publication year, the number of patients with schizophrenia that had been included in the study, information about the exact methodology that had been used, the risk of bias, the kind of treatment intervention, the details of the control interventions (if any), and, finally, the effects of acupuncture treatment on the patient(s) with schizophrenia and the conclusions that had been drawn by the authors of the study. In cases of disagreement, two different authors (S.L. and S.H.L.) were asked to evaluate the study in question for inclusion in this review. In the end, in all cases, consensus was reached among all six authors.

## 3. Results

**Figure 1:**
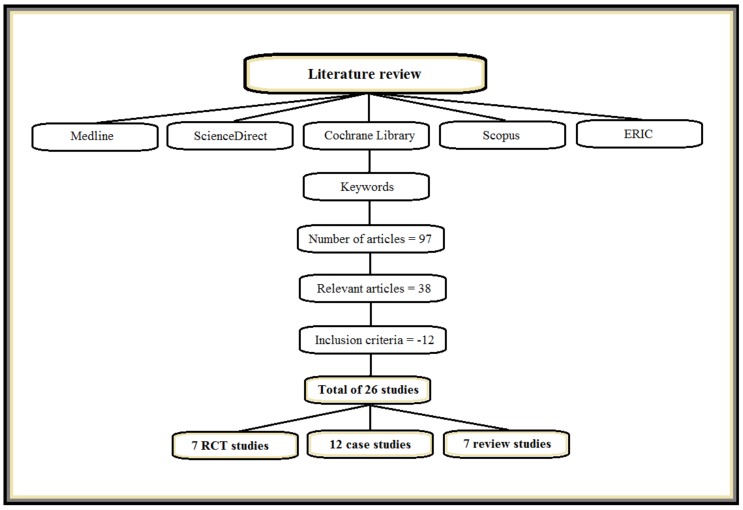
Overview of the study selection.

As can be seen in Figure 1, our search resulted in 97 articles, of which 38 articles were relevant, but only 26 of those satisfied the inclusion criteria and were, thus, eligible for inclusion in this review. Of the 26, 7 were randomized controlled trials [32,33,34,35,36,37,38], 12 were case studies [39,40,41,42,43,44,45,46,47,48,49,50], and 7 were review studies [51,52,53,54,55,56,57]. The total number of patients with schizophrenia that received acupuncture treatment in all randomized controlled trials and case studies together was *n* = 1181. With respect to the demographic background of the acupuncture research on schizophrenia, the studies were conducted on several continents, with 9 (34.62%) having been conducted in Asia (e.g., China, Korea, and Japan) (e.g., [35,36,39,40,41,42]), 13 (50%) in Europe (e.g., Germany, The Netherlands, United Kingdom, and Belgium), 2 (7.69%) in the Middle East (Israel) [50,51], 1 (3.85%) in Africa (Tunisia) [34], and 1 (3.85%) in the United States. However, of the 13 studies done in Europe, only one (1/13 = 7.69%) was conducted solely in Europe [46]. The other 12 (12/13 = 92.31%) were European/Asian collaborations with China or Korea as a partner (e.g., [32,33,37,56]). Remarkably, only one old study from 1979 [48] on acupuncture in the treatment of patients with schizophrenia has been conducted in the USA so far, even though the USA currently plays a key role in worldwide acupuncture research followed by China (e.g., [58,59]).

### 3.1. Safety Issue

Patients with schizophrenia are a very vulnerable patient group [60], so exploring what is known about the safety of using acupuncture in this specific patient group is an important first step. As mentioned before, these patients suffer from delusions and hallucinations [61]. As such, these patients can easily perceive the acupuncture needles differently, i.e., they could include them in paranoid thoughts, think that people can control and register their thoughts via the needles, etc. To guarantee the safety of the patients, group acupuncture therapy in which an acupuncturist is always present is often used instead of individual acupuncture [32]. Serious side-effects of acupuncture treatment have not been reported in the literature [62]. MacPherson and colleagues conducted several studies (e.g., [62,63,64]) on the safety aspect of acupuncture treatment in general and found it to be a safe treatment, as long as the acupuncture was conducted by regulated practitioners [64]. No serious side-effects have been found [63], and in about 1% of the treatments, minor adverse side-effects, such as mild bruising, pain, and bleeding, have been reported [62]. Moreover, the use of acupuncture to treat patients with schizophrenia was reported to be safe in the literature [33]. Although anxiety exists about providing acupuncture treatment to patients with schizophrenia, especially in the West, in the present review, no evidence was found that those patients cannot be safely treated with acupuncture as long as precautionary measures (e.g., always one therapist present and group therapy) are taken into account; moreover, the use of needles did not seem to evoke any negative emotional reactions.

### 3.2. Positive and Negative Symptoms

Overall, most studies showed limited evidence for the use of acupuncture as add-on therapy in the treatment of the positive and negative symptoms [34,54,55,56,57]. However, it is important to note the distinction between quantitative and qualitative results. As Bosch and colleagues [43] explained in their study, a patient with schizophrenia might still suffer from hallucinations (i.e., meaning no quantitative improvement on a symptom checklist, for instance, because the hallucinations still exist), but might be less disturbed because of a difference in severity (i.e., a qualitative improvement). In their study, Wu and Bi [49] also reported five cases where an alleviation of the positive symptoms occurred, but the auditory hallucinations did not disappear completely. Moreover, Xu and colleagues found [36] that, although acupuncture treatment combined with small doses of antipsychotics had the same efficacy as treatment with full-doses of antipsychotics, the use of small doses had important advantages: the initiation time of the acupuncture treatment combined with small doses of antipsychotics was shorter than that of treatment with full-doses of antipsychotics, and the side effects were fewer. In line with the previous finding, Ronan and colleagues [46] reported fewer side effects of the medication in patients with schizophrenia after add-on acupuncture treatment had been started, and Tani and colleagues [47] reported a positive effect of acupuncture treatment on tardive dystonia in a patient with schizophrenia receiving pharmacological treatment. Importantly, Kane and Di Scipio [48] refer in their study to the diagnostic problem of the heterogeneity of patients with schizophrenia. The authors discussed mixed positive results of acupuncture treatment in three patients with schizophrenia: positive treatment results were found for the two patients who had had florid schizophrenic symptoms, whereas no significant response to acupuncture treatment was found for the patient with primarily affective-depressive symptoms.

An exception to the general finding of limited evidence for the use of acupuncture as an additional intervention in the treatment of the clinical symptoms of patients suffering from schizophrenia are the results reported in several acupuncture case studies conducted in China in the 1980s [39,40,41,42]. Those studies reported beneficial acupuncture effects in the treatment of, especially, positive symptoms, such as hallucinations, in patients with schizophrenia [40,41,42]. Shi [42] found that particularly auricular acupuncture (i.e., on the ear) was effective in treating the auditory hallucinations of patients suffering from schizophrenia.

### 3.3. Cognitive Symptoms

Several acupuncture studies included data on cognitive tasks for patients with schizophrenia [33,44]. In most cases, behavioral data on the so-called executive functioning tasks, such as working memory tests, were reported. In a case study, an improved performance in working memory performance immediately after finishing the 12 weeks of acupuncture treatment that remained stable at follow-up, three months later, involving a patient with schizophrenia was reported [44]. Moreover, Bosch and colleagues [33] conducted a pragmatic clinical trial on 50 patients with schizophrenia. Again, all patients were tested on two different working memory tasks before and after 12 weeks of acupuncture treatment. In contrast to the previously discussed case report [44], however, no beneficial effect of acupuncture was found on the working memory performance of patients with schizophrenia.

### 3.4. Sleep Disorders

Positive results have been reported for the treatment of the accompanying sleep disorders [35,51]. For instance, Reshef and colleagues [50] reported a beneficial effect of eight weeks of acupuncture treatment (twice a week) in 20 patients suffering from schizophrenia. A significant improvement after acupuncture treatment was found for numerous sleep variables (e.g., sleep onset latency, sleep percentage, mean activity level, wake time after sleep onset, mean number of wake episodes, mean wake episode and longest wake episode) as measured with objective (actigraphy) sleep measurements. Bosch and colleagues also found beneficial effects of acupuncture treatment on sleep in several case studies of long-term patients suffering from schizophrenia and accompanying sleep disorders on both subjective sleep measurements (questionnaires) [43] as well as on objective (actigraphy) sleep measurements [44,45]. However, those positive effects of acupuncture in the treatment of the accompanying sleep disorders could not be replicated in a randomized, controlled trial on 40 outpatients with schizophrenia in which solely subjective sleep measurements (questionnaires) were used [38]. Huang and Zheng [35] conducted a randomized, controlled trial on 96 patients with schizophrenia and accompanying sleep disorders by using subjective sleep measurements. They found a positive effect of acupuncture that was similar to that of the sleep medication eszopiclone, but treatment using acupuncture was safer than treatment using eszopiclone. Moreover, the percentage of drop-outs was slightly better in the acupuncture group compared to the sleep medication group (2.08% versus 4.17%).

In acupuncture research on patients with schizophrenia and accompanying sleep disorders, both objective and subjective sleep measurements are being used with mixed results [45,50]. Whereas Reshef and colleagues [50] reported better acupuncture effects on the objective sleep measurements than on the subjective sleep measurements, Bosch and colleagues, on the contrary, reported better results on the subjective sleep measurements than on the objective sleep measurements [45].

## 4. Discussion

A systematic review was conducted on the use of acupuncture as an add-on treatment for patients with schizophrenia, with a special focus on the treatment of the often accompanying sleep disorders in this patient group. Many studies so far have found that acupuncture can be safely applied as an additional treatment technique, as long as precautionary measures (e.g., always one therapist present and group therapy) are taken [32]. No serious side effects of acupuncture treatment have been reported in the literature, nor does the use of needles seem to evoke any negative emotional reactions [32]. This is an important finding because, in clinical practice, one of the objections against using acupuncture as an add-on treatment is the idea that in such a vulnerable patient group as patients with schizophrenia it is not safe. A common perception is that patients with schizophrenia who suffer from delusions and hallucinations could perceive the acupuncture needles differently than others: for instance, they might include them in their psychotic thoughts, which might lead to even more positive symptoms. The results of the present review, however, show that acupuncture can be applied safely in this vulnerable patient group [33].

Secondly, in the present review, only limited evidence was found for the use of acupuncture as add-on therapy in the treatment of clinical symptoms [54,55,56,57]. Note that these more recent results are in sharp contrast with the results reported in several acupuncture case studies conducted in China in the 1980s [39,40,41,42]. Those studies reported beneficial effects of acupuncture in the treatment of patients with schizophrenia, especially the treatment of positive symptoms such as hallucinations [40,41,42]. The reason for this discrepancy in results might be the weaker methodology that was used in those older Chinese studies (e.g., no clinical inventories were used, and all data were based on case studies and not on randomized, controlled trials). With respect to the limited evidence for acupuncture’s having an effect on the clinical symptoms, an important observation with respect to positive symptoms is that a patient might score the same on a clinical inventory, even though a qualitative difference may exist at the same [43]; for example, the patient with schizophrenia still suffers from hallucinations, but the severity of the hallucination differs and becomes less disturbing (i.e., a qualitative improvement). In future research, this methodological issue should be investigated more closely to be better able to measure this qualitative result.

With respect to the cognitive symptoms, patients with schizophrenia do not seem to benefit from acupuncture treatment. Although an immediate and sustained positive effect of acupuncture treatment was found in a case study [44], that result was not found in a larger pragmatic clinical trial [33]. However, because only a few studies have been conducted on the possible effect of acupuncture treatment on the cognitive symptoms, more future studies are warranted before any strong conclusions can be drawn.

Finally, the present review shows that positive results have been reported in the literature for the treatment of the accompanying sleep disorders of patients with schizophrenia [35,43,44,45,50]. For instance, the following sleep variables, i.e., sleep onset latency, sleep percentage, mean activity level, wake time after sleep onset, mean number of wake episodes, mean wake episode and longest wake episode, have been found to be significantly improved after acupuncture treatment [50]. Moreover, Huang and Zheng [35] found a positive effect of acupuncture that was similar to that of the sleep medication eszopiclone, but acupuncture was found to be safer than using eszopiclone. However, these beneficial effects of acupuncture treatment in patients with schizophrenia and accompanying sleep disorders are not always found, as Bosch and colleagues [38] could not replicate these findings. In some studies, better results are found on the objective sleep measurements compared to the subjective sleep measurements [50], whereas, in other studies, the opposite result is found [45]. In sum, several studies report beneficial effects of acupuncture in the treatment of the sleep disorders in patients with schizophrenia [35,43,44,45,50]; however, more randomized, controlled trials are needed to replicate this finding and to shed light on the question as to whether objective or subjective sleep measurements give more reliable indications of the success of the treatment.

**Figure 2:**
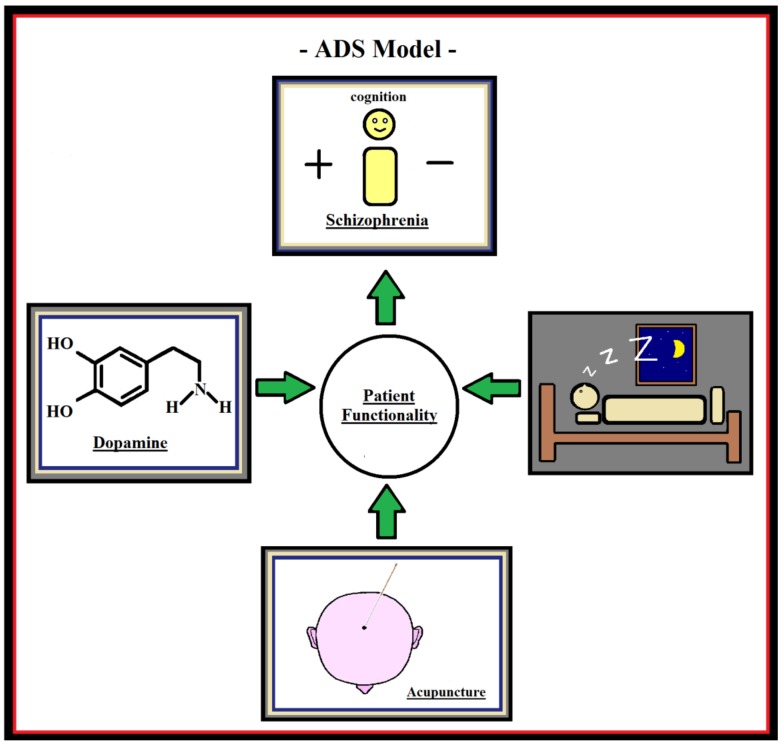
The ADS-model. Acupuncture is able to normalize the dopamine system, which is known to play a role in sleep regulation; thus, by improving sleep, acupuncture is beneficial for a patient suffering from schizophrenia and accompanying sleep disorders.

We propose testing a hypothesis/working model, which we call the “Acupuncture–Dopamine–Sleep” (ADS) hypothesis, in future research (Figure 2). Previous research has shown neuroprotective effects of acupuncture on the dopamine system [65]; moreover, a relation has been found to exist between dopamine and sleep regulation (affecting the circadian rhythm in humans) [66] and between dopamine and schizophrenia [67], which is often referred to as the dopamine hypothesis in the literature. According to the dopamine hypothesis, symptoms in schizophrenia, such as psychoses, are the result of a disturbed and hyperactive dopamine system (for a detailed discussion of the first, second, and third versions of the dopamine hypothesis in schizophrenia, we refer the readers to Howes and Kapur [68]). The neurotransmitter dopamine acts in the pineal gland, enabling the brain to adapt to light and dark cycles, which are needed for good sleep [66]. The ADS model states that the reason most patients with schizophrenia report better sleep after acupuncture treatment is because the acupuncture treatment affects the disturbed and hyperactive dopamine system in patients with schizophrenia, bringing it back into balance. As a result of the improved sleep, the patients start to feel and function better, without necessarily getting rid of all their clinical symptoms. This indirect working mechanism of acupuncture via sleep might also explain the better results often reported in the literature on acupuncture add-on treatment in patients with depression [53,69,70] because patients with depression are known to suffer even more from sleep disorders than patients with schizophrenia. In future research, the ADS model should be tested further by studying patients with insomnia. Here, the use of longer acupuncture treatment protocols, with several follow-up measurements and both subjective and objective sleep (especially melatonin) measurements, is important. Moreover, animal studies seem to be a promising direction to go to directly test the dopamine–sleep [71] and dopamine–schizophrenia [72] relationship in the proposed ADS model.

Several methodological limitations exist in the research on the efficiency of acupuncture in the treatment of patients with schizophrenia and accompanying sleep disorders. A distinction can be made between limitations that are generic to the particular patient population, in our case patients with schizophrenia, and limitations that are specific to acupuncture research. First, we will discuss limitations that are typical for this specific patient group. For instance, most patients with schizophrenia and accompanying sleep disorders receive intensive pharmacological treatment with benzodiazepines, zolpidem, zaleplon, etc. [73], and large individual differences exist in the kinds of medications [53,74] and in the doses of those medications [53,74] that the patients receive. This negatively influences the acupuncture research on the efficiency of acupuncture as an add-on treatment because all kinds of effects interact with each other [53]. Note that most patients receive one or more different kinds of medications and that the dosages differ [75]. For ethical reasons, of course, the combination and the amount of the pharmacological treatment for a patient with schizophrenia is not allowed to be stopped and/or changed simply to be able to better investigate the effects of acupuncture [53]. However, those problems are part of clinical research in daily clinical practice, and although they form a weakness in the research on the efficiency of acupuncture in this patient group on the one hand, they provide a realistic picture of the daily clinical routine on the other hand. The health of the patients is and should always be the top priority; therefore, although those methodological problems cannot be totally solved, the best methodological option possible should be selected.

Another typical methodological limitation of conducting acupuncture research on patients with schizophrenia and accompanying sleep disorders that is generic to the particular patient population is the heterogeneity of this patient group [76,77], which makes investigating the efficacy and developing the most optimal treatment difficult. This forms a problem when clinically described according to Western medicine diagnostics [61,78], as well as when clinically described according to Eastern medicine diagnostics [37]. In the acupuncture research literature, this methodological problem is referred to as “The Fruit-Basket Problem” [79]. It states that, although the patients have the same Western diagnosis “schizophrenia” according to Western medicine, they have different Eastern medicine diagnostic patterns [79] at the same time. From an Eastern medicine point of view, some patterns are more severe than others; therefore, that acupuncture treatment results differ between patients should not be a surprise [79]. Although fully tackling this methodological problem in clinical acupuncture research on patients with schizophrenia and accompanying sleep disorders is difficult, a recommendation is that at least, the different Eastern medicine diagnostic patterns be mentioned along with the Western medicine diagnosis/diagnoses. In the case of a large clinical sample, the efficacy of acupuncture treatment could be investigated in a better way by analyzing the data according to the different Eastern medicine diagnostic patterns, as well.

A third methodological limitation that is specific to the particular patient population is the fact that in the past, solely subjective sleep measurements have been used in acupuncture research on patients with schizophrenia. In recent years, more objective measurements have been used in research on acupuncture, schizophrenia, and sleep disorders [50]. In addition to the usual standard clinical symptoms/sleep inventories, such as the Positive and Negative Syndrome Scale (PANSS) [80], Pittsburgh Sleep Quality Index (PSQI) [81], and the Epworth Sleepiness Scale [82], objective measurements with, for instance, actiwatches have been conducted [44,45,50]. In future research, the use of melatonin measurements [83] and polysomnography [83] seem to be promising new directions to establish objective sleep measurements. These additional objective measurements will shed more light on the efficiency and the underlying working mechanisms of acupuncture in the treatment of patients with schizophrenia and accompanying sleep disorders.

A limitation that is specific to acupuncture research is the fact that gender has recently become an important issue in acupuncture research [84], and the psychophysiological and neural effects of acupuncture treatment for males has been found to be different from those of acupuncture treatment for females [85]. Females reported a greater intensity of aching compared to males after acupuncture; moreover, females exhibited greater brain activation in many brain areas, i.e., in the rightsided postcentral gyrus, precentral gyrus, precuneus, postcentral gyrus, inferior parietal lobule, declive, middle occipital gyrus and parahippocampal gyrus, relative to men, after acupuncture [85,86]. However, so far, the gender issue seems to have been ignored in clinical research on the efficiency of acupuncture in the treatment of patients with schizophrenia. Future studies on this topic are warranted to develop optimal acupuncture treatments for both male and female patients with schizophrenia suffering from accompanying sleep disorders.

Another limitation of the present study that is specific to acupuncture research is that although the majority of the more recently published acupuncture studies on schizophrenia that were included in the present review are at moderate risk of bias [56], those risks are higher for the few acupuncture studies [39,40,41,42] that were conducted in China in the 1980s [55]. We included as many acupuncture studies on schizophrenia as possible to be able to provide the most complete overview of this developing research field, but, at the same time, we have chosen to treat the conclusions drawn based on those older studies with caution.

A third limitation of our study that is specific to acupuncture research is that, although we have seen an increase in the number of acupuncture studies on patients with schizophrenia during the last years, the overall number of randomized, controlled studies is still low (e.g., seven in the present review). In total, we included 26 studies in the present review, but more future studies, particularly larger and better designed, randomized, controlled, clinical trials, on schizophrenia are needed [52] before any firm conclusions can be drawn on the efficiency of acupuncture as an add-on treatment for patients with schizophrenia suffering from accompanying sleep disorders. In the present review, it was chosen to include only studies that used (mechanical) acupuncture and other modalities, such as electro-acupuncture [29], laser-acupuncture [30], and acupressure [31], were excluded to be able to compare the study results. In future research, it might be interesting to conduct separate review studies on those different acupuncture modalities in the treatment of patients with schizophrenia as well and compare those results with the results found in the present study.

Finally, consensus should be achieved in the acupuncture field on how to scientifically present the TCM diagnostics and acupuncture points used. To date, as we also discovered in the present study, information is often lacking on what TCM protocol was applied, which acupuncture points were exactly used, and, as a result, studies are difficult to compare and to replicate.

## 5. Conclusions

We found limited evidence for the use of acupuncture as add-on therapy in the treatment of patients with schizophrenia; however, positive results for the use of acupuncture were found in the treatment of the accompanying sleep disorders from which patients with schizophrenia often suffer, but this result needs to be confirmed in future large, randomized, controlled trials.

## References

[1] Van Os J., Kapur S. (2009). Schizophrenia. Lancet.

[2] Iacono W.G., Beiser M. (1992). Are males more likely than females to develop schizophrenia?. Am. J. Psychiatry.

[3] McGrath J., Saha S., Welham J., El Saadi O., MacCauley C., Chant D. (2004). A systematic review of the incidence of schizophrenia: The distribution of rates and the influence of sex, urbanicity, migrant status and methodology. BMC Med..

[4] Picchioni M.M., Murray R.M. (2007). Schizophrenia. BMJ.

[5] Castle D., Wessely S., Der G., Murray R.M. (1991). The incidence of operationally defined schizophrenia in Camberwell, 1965–1984. Br. J. Psychiatry.

[6] Kelly B.D., O’Callaghan E., Waddington J.L., Feeney L., Browne S., Scully P.J., Clarke M., Quinn J.F., McTigue O., Morgan M.G. (2010). Schizophrenia and the city: A review of literature and prospective study of psychosis and urbanicity in Ireland. Schizophr. Res..

[7] Carbon M., Correll C.U. (2014). Thinking and acting beyond the positive: The role of the cognitive and negative symptoms in schizophrenia. CNS Spectr..

[8] Teeple R.C., Caplan J.P., Stern T.A. (2009). Visual hallucinations: Differential diagnosis and treatment. Prim. Care Companion J. Clin. Psychiatry.

[9] Foussias G., Remington G. (2010). Negative symptoms in schizophrenia: Avolition and Occam’s razor. Schizophr. Bull..

[10] Orellana G., Slachevsky A. (2013). Executive functioning in schizophrenia. Front. Psychiatry.

[11] Gruber O., Chadha Santuccione A., Aach H. (2014). Magnetic resonance imaging in studying schizophrenia, negative symptoms, and the glutamate system. Front. Psychiatry.

[12] De Hert M., McKenzie K., Peuskens J. (2001). Risk factors for suicide in young people suffering from schizophrenia: A long-term follow-up study. Schizophr. Res..

[13] Kane J.M., Correll C.U. (2010). Pharmacologic treatment of schizophrenia. Dialogues Clin. Neurosci..

[14] Jaffe A.B., Levine J. (2003). Efficacy and effectiveness of first- and second-generation antipsychotics in schizophrenia. J. Clin. Psychiatry.

[15] Smith M., Hopkins D., Peveler R.C., Holt R.I., Woodward M., Ismail K. (2008). First- v. second-generation antipsychotics and risk for diabetes in schizophrenia: Systematic review and meta-analysis. Br. J. Psychiatry.

[16] Miller D.D., Caroff S.N., Davis S.M., Rosenheck R.A., McEvoy J.P., Saltz B.L., Riggio S., Chakos M.H., Swartz M.S., Keefe R.S. (2008). Extrapyramidal side-effects of antipsychotics in a randomised trial. Br. J. Psychiatry.

[17] Correll C.U., Schenk E.M. (2008). Tardive dyskinesia and new antipsychotics. Curr. Opin. Psychiatry.

[18] Krebs M., Leopold K., Hinzpeter A., Schaefer M. (2006). Current schizophrenia drugs: Efficacy and side effects. Expert Opin. Pharmacother..

[19] Lieberman J.A. (2004). Metabolic changes associated with antipsychotic use. Prim. Care Companion J. Clin. Psychiatry.

[20] Kemmler G., Hummer M., Widschwendter C., Fleischhacker W.W. (2005). Dropout rates in placebo-controlled and active-control clinical trials of antipsychotic drugs: A meta-analysis. Arch. Gen. Psychiatry.

[21] Zhang J.P., Malhotra A.K. (2011). Pharmacogenetics and antipsychotics: Therapeutic efficacy and side effects prediction. Expert Opin. Drug Metab. Toxicol..

[22] Dickerson F.B., Lehman A.F. (2006). Evidence-based psychotherapy for schizophrenia. J. Nerv. Ment. Dis..

[23] Kopelowicz A., Liberman R.P., Zarate R. (2006). Recent advances in social skills training for schizophrenia. Schizophr. Bull..

[24] Waite F., Myers E., Harvey A.G., Espie C.A., Startup H., Sheaves B., Freeman D. (2016). Treating sleep problems in patients with schizophrenia. Behav. Cogn. Psychother..

[25] Caqueo-Urízar A., Rus-Calafell M., Urzúa A., Escudero J., Gutiérrez-Maldonado J. (2015). The role of family therapy in the management of schizophrenia: Challenges and solutions. Neuropsychiatr. Dis. Treat..

[26] Twamley E.W., Jeste D.V., Lehman A.F. (2003). Vocational rehabilitation in schizophrenia and other psychotic disorders: A literature review and meta-analysis of randomized controlled trials. J. Nerv. Ment. Dis..

[27] Dey T. (1999). Soothing the Troubled Mind: Acupuncture and Moxibustion in the Treatment and Prevention of Schizophrenia.

[28] Moher D., Liberati A., Tetzlaff J., Altman D.G., The PRISMA Group (2009). Preferred reporting items for systematic reviews and meta-analyses: The PRISMA statement. BMJ.

[29] Cheng J., Wang G., Xiao L., Wang H., Wang X., Li C. (2009). Electro-acupuncture versus sham electro-acupuncture for auditory hallucinations in patients with schizophrenia: A randomized controlled trial. Clin. Rehabil..

[30] Zhang B. (1991). A controlled study of clinical therapeutic effects of laser acupuncture for schizophrenia. Zhonghua Shen Jing Jing Shen Ke Za Zhi.

[31] Ching H.Y., Wu S.L., Chen W.C., Hsieh C.L. (2012). Effects of auricular acupressure on body weight parameters in patients with chronic schizophrenia. Evid. Based Complement. Altern. Med..

[32] Bosch P., van Luijtelaar G., van den Noort M., Lim S., Egger J., Coenen A. (2013). Sleep ameliorating effects of acupuncture in a psychiatric population. Evid. Based Complement. Altern. Med..

[33] Bosch P., van den Noort M., Yeo S., Lim S., Coenen A., van Luijtelaar G. (2015). The effect of acupuncture on mood and working memory in patients with depression and schizophrenia. J. Integr. Med..

[34] Bouhlel S., El-Hechmi S., Ghanmi L., Ghaouar M., Besbes C., Khaled M., Melki W., El-Hechmi Z. (2011). Effectiveness of acupuncture in treating schizophrenia: A clinical randomized trial of 31 patients. Tunis Med..

[35] Huang Y., Zheng Y. (2015). Sleep disorder of schizophrenia treated with shallow needling: A randomized controlled trial. Zhongguo Zhen Jiu.

[36] Xu T.C., Su J., Wang W.N. (2010). Effect of three-step acupuncture combined with small dosage antipsychotic in treating incipient schizophrenia. Zhongguo Zhong Xi Yi Jie He Za Zhi.

[37] Bosch P., de Rover P., Staudte H., Lim S., van den Noort M. (2015). Schizophrenia, depression, and sleep disorders: Their traditional Oriental medicine equivalents. J. Acupunct. Meridian Stud..

[38] Bosch P., van den Noort M., Staudte H., Lim S., Yeo S., Coenen A., van Luijtelaar G. (2016). Sleep disorders in patients with depression or schizophrenia: A randomized controlled trial using acupuncture treatment. Eur. J. Integr. Med..

[39] Shi Z.X., Tan M.Z. (1986). An analysis of the therapeutic effect of acupuncture treatment in 500 cases of schizophrenia. J. Tradit. Chin. Med..

[40] Zhang M.J. (1988). Treatment of 296 cases of hallucination with scalp-acupuncture. J. Tradit. Chin. Med..

[41] Shi Z.X. (1988). Observation on the therapeutic effect of 120 cases of hallucination treated with auricular acupuncture. J. Tradit. Chin. Med..

[42] Shi Z.X. (1989). Observation on the curative effect of 120 cases of auditory hallucination treated with auricular acupuncture. J. Tradit. Chin. Med..

[43] Bosch P., Staudte H., van den Noort M., Lim S. (2014). A case study on acupuncture in the treatment of schizophrenia. Acupunct. Med..

[44] Bosch P., Lim S., Yeo S., Lee S.H., Staudte H., van den Noort M. (2016). Acupuncture in the treatment of a female patient suffering from chronic schizophrenia and sleep disorders. Case Rep. Psychiatry.

[45] Bosch P., Staudte H., Yeo S., Lee S.H., Lim S., van den Noort M. (2017). Acupuncture treatment of a male patient suffering from long-term schizophrenia and sleep disorders. J. Tradit. Chin. Med..

[46] Ronan P., Robinson N., Harbinson D., MaxInnes D. (2011). A case study exploration of the value of acupuncture as an adjunct treatment for patients diagnosed with schizophrenia: Results and future study design. Zhong Xi Yi Jie He Xue Bao.

[47] Tani M., Suzuki T., Takada A., Yagyu T., Kinoshita T. (2005). Effect of acupuncture treatment for a patient with severe axial dystonia appearing during treatment for schizophrenia. Seishin Shinkeigaku Zasshi.

[48] Kane J., Di Scipio W.J. (1979). Acupuncture treatment of schizophrenia: Report on three cases. Am. J. Psychiatry.

[49] Wu Y., Bi S. (2004). Combined use of acupuncture and pharmacotherapy for treatment of auditory hallucination. J. Tradit. Chin. Med..

[50] Reshev A., Bloch B., Vadas L., Ravid S., Kremer I., Haimov I. (2013). The effects of acupuncture treatment on sleep quality and on emotional measures among individuals living with schizophrenia: A pilot study. Sleep Disord..

[51] Bloch B., Ravid S., Vadas L., Reshev A., Schiff E., Kremer I., Haimov I. (2010). The acupuncture treatment of schizophrenia: A review with case studies. J. Chin. Med..

[52] Beecroft N., Rampes H. (1997). Review of acupuncture for schizophrenia. Acupunct. Med..

[53] Bosch P., van den Noort M., Staudte H., Lim S. (2015). Schizophrenia and depression: A systematic review of the effectiveness and the working mechanisms behind acupuncture. Explore.

[54] Rathbone J., Xia J. (2005). Acupuncture for schizophrenia. Cochrane Database Syst. Rev..

[55] Lee M.S., Shin B.C., Ronan P., Ernst E. (2009). Acupuncture for schizophrenia: A systematic review and meta-analysis. Int. J. Clin. Pract..

[56] Shen X., Xia J., Adams C.E. (2014). Acupuncture for schizophrenia. Cochrane Database Syst. Rev..

[57] Shen X., Xia J., Adams C. (2014). Acupuncture for schizophrenia. Schizophr. Bull..

[58] Maeda Y., Kim H., Kettner N., Kim J., Cina S., Malatesta C., Gerber J., McManus C., Ong-Sutherland R., Mezzacappa P. (2017). Rewiring the primary somatosensory cortex in carpal tunnel syndrome with acupuncture. Brain.

[59] Han J.S., Ho Y.S. (2011). Global trends and performances of acupuncture research. Neurosci. Biobehav. Rev..

[60] Viron M., Baggett T., Hill M., Freudenreich O. (2012). Schizophrenia for primary care providers: How to contribute to the care of a vulnerable patient population. Am. J. Med..

[61] American Psychiatric Association (2013). Diagnostic and Statistical Manual of Mental Disorders.

[62] MacPherson H., Thomas K., Walters S., Fitter M. (2001). The York acupuncture safety study: Prospective survey of 34,000 treatments by traditional acupuncturists. BMJ.

[63] MacPherson H., Thomas K., Walters S., Fitter M. (2001). A prospective survey of adverse events and treatment reactions following 34,000 consultations with professional acupuncturists. Acupunct. Med..

[64] MacPherson H., Scullion A., Thomas K.J., Walters S. (2004). Patient reports of adverse events associated with acupuncture treatment: A prospective national survey. Qual. Saf. Health Care.

[65] Kang J.M., Park H.J., Choi Y.G., Choe I.H., Park J.H., Kim Y.S., Lim S. (2007). Acupuncture inhibits microglial activation and inflammatory events in the MPTP-induced mouse model. Brain Res..

[66] González S., Moreno-Delgado D., Moreno E., Pérez-Capote K., Franco R., Mallol J., Cortés A., Casadó V., Lluís C., Ortiz J. (2012). Circadian-related heteromerization of adrenergic and dopamine D_4_ receptors modulates melatonin synthesis and release in the pineal gland. PLoS Biol..

[67] Seeman P., Kapur S. (2000). Schizophrenia: More dopamine, more D_2_ receptors. Proc. Natl. Acad. Sci. USA.

[68] Howes O.D., Kapur S. (2009). The dopamine hypothesis of schizophrenia: Version III—The final common pathway. Schizophr. Bull..

[69] Chan Y.Y., Lo W.Y., Yang S.N., Chen Y.H., Lin J.G. (2015). The benefit of combined acupuncture and antidepressant medication for depression: A systematic review and meta-analysis. J. Affect. Disord..

[70] Wang H., Qi H., Wang B.S., Cui Y.Y., Zhu L., Rong Z.X., Chen H.Z. (2008). Is acupuncture beneficial in depression: A meta-analysis of 8 randomized controlled trials?. J. Affect. Disord..

[71] Lim M.M., Xu J., Holtzman D.M., Mach R.H. (2011). Sleep deprivation differentially affects dopamine receptor subtypes in mouse striatum. Neuroreport.

[72] Rung J.P., Carlsson A., Markinhuhta K.R., Carlsson M.L. (2005). The dopaminergic stabilizers (−)-OSU6162 and ACR16 reverse (+)-MK-801-induced social withdrawal in rats. Prog. Neuropsychopharmacol. Biol. Psychiatry.

[73] Van den Noort M., Staudte H., Perriard B., Yeo S., Lim S., Bosch P. (2016). Schizophrenia and comorbid sleep disorders. Neuroimmunol. Neuroinflamm..

[74] Ren X.S., Kazis L.E., Lee A.F., Hamed A., Huang Y.H., Cunningham F., Miller D.R. (2002). Patient characteristics and prescription patterns of atypical antipsychotics among patients with schizophrenia. J. Clin. Pharm. Ther..

[75] Tsutsumi C., Uchida H., Suzuki T., Watanabe K., Takeuchi H., Nakajima S., Kimura Y., Tsutsumi Y., Ishii K., Imasaka Y. (2011). The evolution of antipsychotic switch and polypharmacy in natural practice: A longitudinal perspective. Schizophr. Res..

[76] McGrath J. (2008). Dissecting the heterogeneity of schizophrenia outcomes. Schizophr. Bull..

[77] Jablensky A. (2015). Schizophrenia or schizophrenias? The challenge of genetic parsing of a complex disorder. Am. J. Psychiatry.

[78] World Health Organization (1992). The ICD-10 Classification of Mental and Behavioural Disorders: Clinical Descriptions and Diagnostic Guidelines.

[79] Bosch P., de Rover P., Yeo S., Lee S.H., Lim S., van den Noort M. (2016). Traditional Chinese medicine in psychiatry: The fruit-basket-problem. J. Integr. Med..

[80] Kay S.R., Fiszbein A., Opler L.A. (1987). The positive and negative syndrome scale (PANSS) for schizophrenia. Schizophr. Bull..

[81] Buysse D.J., Reynolds C.F., Monk T.H., Berman S.R., Kupfer D.J. (1989). The Pittsburgh Sleep Quality Index: A new instrument for psychiatric practice and research. Psychiatry Res..

[82] Johns M.W. (1991). A new method for measuring daytime sleepiness: The Epworth sleepiness scale. Sleep.

[83] Rahman S.A., Kayumov L., Tchmoutina E.A., Shapiro C.M. (2009). Clinical efficacy of dim light melatonin onset testing in diagnosing delayed sleep phase syndrome. Sleep Med..

[84] Lee S.H., van den Noort M., Bosch P., Lim S. (2016). Sex differences in acupuncture effectiveness in animal models of Parkinson’s disease: A systematic review. BMC Complement. Altern. Med..

[85] Yeo S., Rosen B., Bosch P., van den Noort M., Lim S. (2016). Gender differences in the neural response to acupuncture: Clinical implications. Acupunct. Med..

[86] Litscher D., Wang J., Litscher G., Li G., Bosch P., van den Noort M., Wang L. (2018). Gender differences in laser-acupuncture results of a crossover study with green and yellow laser at the ear point Shenmen. Medicines.

